# Increased Virulence of *Culicoides* Midge Cell-Derived Bluetongue Virus in IFNAR Mice

**DOI:** 10.3390/v16091474

**Published:** 2024-09-17

**Authors:** Barbara S. Drolet, Lindsey Reister-Hendricks, Christie Mayo, Case Rodgers, David C. Molik, David Scott McVey

**Affiliations:** 1 Arthropod-Borne Animal Diseases Research Unit, Agricultural Research Service, United States Department of Agriculture, Manhattan, KS 66502, USA; lindsey.reister-hendricks@usda.gov (L.R.-H.); david.molik@usda.gov (D.C.M.); 2 Department of Microbiology, Immunology, and Pathology, Colorado State University, 1601 Campus Delivery, Fort Collins, CO 80526, USA; christie.mayo@colostate.edu (C.M.); case1prod@gmail.com (C.R.); 3 School of Veterinary Medicine and Biomedical Sciences, University of Nebraska-Lincoln, P.O. Box 830905, Lincoln, NE 68583, USA; dmcvey2@unl.edu

**Keywords:** bluetongue virus, BTV, *Culicoides*, biting midge, IFNAR mice, virulence

## Abstract

Bluetongue (BT) is a *Culicoides* midge-borne hemorrhagic disease affecting cervids and ruminant livestock species, resulting in significant economic losses from animal production and trade restrictions. Experimental animal infections using the α/β interferon receptor knockout IFNAR mouse model and susceptible target species are critical for understanding viral pathogenesis, virulence, and evaluating vaccines. However, conducting experimental vector-borne transmission studies with the vector itself are logistically difficult and experimentally problematic. Therefore, experimental infections are induced by hypodermic injection with virus typically derived from baby hamster kidney (BHK) cells. Unfortunately, for many U.S. BTV serotypes, it is difficult to replicate the severity of the disease seen in natural, midge-transmitted infections by injecting BHK-derived virus into target host animals. Using the IFNAR BTV murine model, we compared the virulence of traditional BHK cell-derived BTV-17 with *C. sonorensis* midge (W8) cell-derived BTV-17 to determine whether using cells of the transmission vector would provide an in vitro virulence aspect of vector-transmitted virus. At both low and high doses, mice inoculated with W8-BTV-17 had an earlier onset of viremia, earlier onset and peak of clinical signs, and significantly higher mortality compared to mice inoculated with BHK-BTV-17. Our results suggest using a *Culicoides* W8 cell-derived inoculum may provide an in vitro vector-enhanced infection to more closely represent disease levels seen in natural midge-transmitted infections while avoiding the logistical and experimental complexity of working with live midges.

## 1. Introduction

Bluetongue (BT) is a vector-borne hemorrhagic disease of cervids and ruminant livestock species resulting in significant economic losses from decreased animal production and non-tariff trade restrictions on animals and animal products. Nearly 30 serotypes of the causative agent, bluetongue virus (BTV), have been reported worldwide [1]. In the U.S., 17 serotypes are either established or reported [2] and all are transmitted solely by *Culicoides* spp. biting midges during blood feeding. In natural infections, the pathology is characterized by widespread edema, hemorrhaging (lymph nodes, lungs, heart, muscles), and necrosis of mucosal surfaces in the oro-nasal and alimentary systems [3,4]. This pathology results in clinical signs to include fever, respiratory distress, nasal and ocular discharge, oral ulcers, coronitis, cyanotic tongues, and death.

Experimental animal infections in the α/β interferon receptor (IFNAR) knockout BTV murine model [5,6,7,8] and in target ruminant species are critical for understanding BTV virulence, pathogenesis, immune responses to infection, and evaluating vaccine candidates [9]. Yet, major gaps exist in understanding the virus-vector-host interactions that underlie transmission efficiency and severity of clinical disease seen in natural infections. While experimentally replicating natural, midge-borne infections is desirable, using midges to deliver the virus is logistically difficult and experimentally problematic. Thus, BTV animal infection experimental designs typically remove the insect from the equation and rely on injecting mammalian cell culture-derived virus or blood from viremic animals.

Apart from the highly virulent European BTV-8 serotype [10,11,12,13,14,15], even when an outbreak results in severe disease of sheep, experimental infections with that field isolate often result in lower viremia and milder clinical disease than that seen in the natural, midge-transmitted infections. Whereas the bite of a single experimentally-infected midge has been shown to result in BTV transmission and clinical disease [16], experimental animal infections by needle inoculation often require unnaturally large volumes of high viral titer blood from an infected animal of the same or different species [13,15,17,18,19] or viral inocula derived from baby hamster kidney (BHK) cells [12,20,21,22,23,24,25,26] to obtain viremia and clinical disease similar to what is seen during outbreaks. Experimental inoculation routes are also problematic. Although intradermal injections are more difficult to perform accurately, this route of exposure most closely mimics midge-borne transmission [15,24]. However, these large inoculum volumes require subcutaneous (SC) injections [13,22,23,25,27,28], intravenous (IV) injections [17,26,29,30], or a combination of these infection routes [12,14,20,21,31,32], which exposes cell types and elicits immune responses atypical of midge-borne infections. 

The enhanced pathology and clinical disease observed with midge-transmitted BTV is likely multifactorial. When a midge ingests virus from an infected animal, it replicates within midge tissues without apparent damage, disseminates to the salivary glands, and is delivered to a susceptible host during the next blood feeding. Thus, midge cell-derived virus populations must quickly adapt to the new host environment and efficiently establish infection in mammalian cells before being cleared by innate immune responses. This efficiency is not clearly understood but may be facilitated by the myriad of salivary proteins delivered along with virus, which enable the midge to feed to repletion and protect its midgut against the host’s innate and adaptive immune responses [33,34]. Midge saliva has also been shown to elicit leukocyte infiltration and inflammatory immune responses in murine dermal tissue that begin locally at the bite site and progress to a systemic response [18]. These responses likely contribute to the increased transmission efficiency, immunopathology, and overall clinical disease seen in susceptible animals when the infection is midge-borne (natural infection) versus needle inoculation (experimental infection).

Natural, midge-borne BTV-17 outbreaks in U.S. sheep result in moderate to severe clinical disease and up to 35% mortality [35]. Yet, using high-titer BHK-derived viral inocula of this and other established U.S. serotypes in experimental infections often results in only a mild, transient fever and falls short on being useful for pathogenesis or vaccine research. We investigated whether using cells of the BTV vector, *Culicoides sonorensis*, to produce an inoculum of BTV-17 would enhance virulence and clinical disease observed in IFNAR mice compared to virus produced in the more traditionally used cell line, BHK. Clinical disease, viremia, tissue distribution of virus, and survival were compared in low and high dose infections.

## 2. Materials and Methods

### 2.1. Cell Lines

Baby hamster kidney cells (BHK-21; ATCC CCL-10), used for producing the BHK-BTV-17 stock virus, were maintained at 37 °C and 5% CO_2_ in Eagles MEM with Earle’s salts (Sigma, St. Louis, MO, USA), 2% FBS, and penicillin/streptomycin sulfate (100 U). *Culicoides sonorensis* cells (W8; Arthropod-Borne Animal Diseases Research Unit, Manhattan, KS, USA) [36], used for producing the W8-BTV-17 stock virus, were maintained at 28 °C in Schneider’s Insect Media (Sigma-Aldrich) (24.5 g/L) supplemented with 0.4 g/L sodium bicarbonate, 0.0585 g/L L-glutamine, 0.006 g/L reduced glutathione, 0.03 g/L L-asparagine, 18 μL of 10 mg/L bovine insulin, and 15% insect-tested FBS. Vero MARU cells (VM; Middle America Research Unit, Panama), used for titrating virus from stocks and mouse blood and tissue samples by plaque assay, were grown in 199E media (Sigma-Aldrich) containing 2% FBS, 100 ug/mL of streptomycin, 100 units/mL penicillin, and 0.25 ug/mL of amphotericin B at 37 °C with 5% CO_2_. 

### 2.2. Bluetongue Virus Serotype 17 (BTV-17)

Two high-titer viral stocks for mouse inoculations were produced with a BTV-17 isolate (514664-179; NVSL, Ames, IA, USA) from the 2007 MT/WY USA outbreak [35]. One stock was propagated in BHK-21 cells (BHK-BTV-17) and one in W8 *Culicoides* cells (W8-BTV-17). Cell monolayers at 80% confluency in 850 cm^2^ roller bottles (BHK) or T-150 flasks (W8) were infected at MOI = 0.1 and allowed to incubate for five days at the conditions above. Virus was harvested by two freeze–thaw cycles followed by centrifugation (Hettich 320R; Grainger, Lenexa, KS, USA) at 1500× *g* for 30 min at 4 °C to pellet cell debris. Cleared viral stock supernatants were titered on Vero MARU cells (as above), aliquoted, and stored at −80 °C. Inocula for mock-infected mice were produced with uninfected BHK and W8 cells processed as above.

### 2.3. Mice 

The BTV-susceptible mouse strain, α/β interferon receptor (IFNAR) knockout, and its BTV-refractory parental mouse strain, C57BL/6J (Jackson Labs, Bar Harbor, ME, USA), were used for all treatments (N = 30/strain). IFNAR (B6.129S2-IFNAR1^tm1Agt^/Mmjax) mice were obtained from breeding colonies at the Comparative Medicine Group (Kansas State University, Manhattan, KS, USA), which were started with one male and three females (Jackson Labs). All mice used were age-matched, female, and weighed 20–30 g. All procedures were approved by Kansas State University Institutional Animal Care and Use Committee (IACUC) under protocol #3659. Mice were acclimated for seven days and caged according to treatment groups and strains. Before inoculation, mice were weighed, the hair from their hind legs and abdomens was removed with shaving followed by depilating cream, and a pre-injection blood sample was obtained from the lateral saphenous vein of a hind leg into EDTA-coated capillary tubes (BD Biosciences, Franklin Lakes, NJ, USA). 

### 2.4. Mice Inoculations 

Following titration of the stock viruses on Vero cells as described above, two injection doses of BHK-BTV-17 and W8-BTV-17 viruses were made by diluting stocks in sterile PBS, pH 7.4. The low dose was 1 × 10^2^ PFU/30 µL (3.35 × 10^3^ PFU/mL) and the high dose titer was 1 × 10^4^ PFU/30 µL (3.35 × 10^5^ PFU/mL). Infectious doses were based on previous BTV IFNAR mouse studies for virulence evaluation and vaccine efficacy trials, resulting in mortality rates of 17% at the low dose and 80–100% at the high dose by 6 days post infection (dpi) [5,7,8].

Mice were anesthetized by intraperitoneal injection of ketamine/xylazine (100 mg/kg:10 mg/kg) [37]. Treatment groups (Table 1) were given a total of six 5 µL transdermal inoculum injections (30 µL total) dispersed bilaterally on their midsection using a 0.5 mL syringe with 30G × 12.7 mm needle (BD Biosciences). Mice recovered on warming pads until mobile, bright, alert, and responsive to stimulation and housed according to treatment and strain in a HEPA-filtered caging system (Lab Products, Inc., Seaford, DE, USA). 

### 2.5. Clinical Disease

Mice were observed daily for health checks and notation of apparent clinical signs consistent with BTV infection. Clinical scores (Table 2) were assigned based on clinical signs including ruffled fur, nasal or ocular discharge, hunched postures, lethargy, labored breathing, and neurological signs such as paralysis, tremors, and ataxia. Observations increased to every 8–12 h for mice with clinical scores of 2 (moderate). Mice were humanely euthanized when disease was rated as severe or if observed weight loss was 20% or greater, in accordance with the approved IACUC protocol.

### 2.6. Sample Collection and Processing

Post-challenge, blood (50 µL) was collected from each mouse daily for 7 days via lateral saphenous vein puncture into EDTA-coated capillary tubes (BD Biosciences) and stored at 4 °C until processed as below. Mice were humanely euthanized during the first 6 days if disease was rated as severe (Table 2; score of 3) or if weight loss was 20% or greater. Upon euthanasia, maximum blood volumes (0.8–1.0 mL) were extracted immediately by cardiac puncture with a 22-gauge needle and syringe.

Tissue samples taken at necropsy included liver, lung, spleen, thymus, and lymph nodes (popliteal, mesenteric, and inguinal). Tissues were placed in 500 µL RNAlater (Thermo Fisher Scientific, Waltham, MA, USA) for subsequent RNA extraction and in 500 µL antibiotic 199E media (containing 2% FBS, 100 µg/mL, streptomycin, 100 units/mL penicillin, and 0.25 µg/mL amphotericin B) for subsequent isolation of infectious virus. Tissues were homogenized by high-speed shaking (Bead Ruptor Elite, Omni-Inc., Kennesaw, GA, USA) for 2 min (3.1 m/s) and centrifuged at 12,000× *g* for 5 min to pellet debris. A 200 µL volume of the cleared homogenate was tested for viral RNA by RT-qPCR as previously described [19] and for infectious virus by standard plaque assay on Vero cells.

### 2.7. Virus Detection

Viral RNA in blood and tissue samples was extracted with MagMAX Total RNA Isolation kits (Thermo Fisher Scientific) and a MagMAX Express Magnetic Particle Processor (Thermo Fisher Scientific) as per manufacturer’s standard automated protocol. RNA was stored in 96-well plates at −80 °C until tested by BTV RT-qPCR as previously described [19]. Briefly, the highly conserved S10 (NS3) and M5 (NS1) genes were detected in BTV RNA and β-actin was used as an internal control [38]. Estimated virus particles were calculated from RNA concentrations as previously described [39]. Blood titers are reported as Log_10_ virus particles per the 50 µL daily sample and tissue titers are reported as Log_10_ viral particles/mL of homogenate. Infectious virus in tissues was titered by standard plaque assay and reported as Log_10_ PFU/mL of homogenate.

### 2.8. Whole Genome Sequencing

Total RNA was extracted [19] from all harvested tissues of one IFNAR mouse in each of the two high dose treatments for viral genome sequencing as previously described [40]. Briefly, sample libraries were prepared using the NEBNext Ultra II Directional RNA Library Prep kit (New England BioLabs Inc., Ipswich, MA, USA). The following modifications were applied to the Section 4 protocol double-stranded RNA: (a) RNA fragmentation was performed at 90 °C for 1 min, and (b) first-strand cDNA synthesis was performed for 10 min at 25 °C, 30 min at 42 °C, and finally, 15 min at 70 °C. Libraries were assessed for DNA quality and concentration with Qubit high sensitivity DNA reagents on the Qubit 2.0 fluorometer (Thermo Fisher Scientific) and High Sensitivity D1000 DNA screentape on a TapeStation 4150 (Agilent Technologies, Santa Clara, CA, USA). A NextSeq 500/550 Mid Output Kit (v2.5) was used to perform 2 × 150 paired-end sequencing on a NextSeq 500 (Illumina, San Diego, CA, USA).

### 2.9. Sequence Analysis

To generate reference sequences for this study, BTV sequences were obtained from the NCBI Sequence Read Archive (SRA) (Table S1), concatenated into a unified file, and each sequence was utilized as a feature in the creation of a genomic feature file in gff3 format, serving as an annotation. The sequence function analysis was executed within the SciNet High-Performance Computing (HPC) environment, leveraging available software, namely FastQC (v0.12.1), MultiQC (v1.21), BBTools (39.01; BBMap, BBDuk), Burrows-Wheeler Aligner (BWA; 0.7.17-r1188), Samtools (1.17), Picard (3.0.0), and Variant Effect Predictor (VEP; 110.1), along with the previously generated reference sequence files. For analysis, raw sequencing data were processed, aligned to the reference genome [41] for variant identification, and subsequently filtered for high-confidence single nucleotide polymorphisms (SNPs). Alignment of reads occurred in two ways: both BHK- and W8-derived BTV-17 genome sequence reads were aligned to the reference genome for comparison, and then the W8-BTV-17 sequences were aligned using BWA and used to create a consensus sequence with Samtools, to which the BHK-BTV-17 sequences were aligned for a comparison between the two viral stocks. 

FastQC was employed to generate comprehensive sequence quality reports, providing quality metrics of the raw sequencing data. Subsequently, MultiQC was utilized to collate and summarize the individual sequence reports, facilitating a consolidated overview of the data. Trimming procedures were executed using BBDuk, informed by the insights gained from the FastQC reports. Alignments of the trimmed reads to the reference sequences were conducted using BWA. Samtools was then used to sort the resultant Binary Alignment Map (BAM) files. Duplicate sequences within the sorted BAM files were identified and marked using Picard tools. The Genome Analysis Toolkit (GATK)-HaplotypeCaller was applied to identify variants for each sample. In the subsequent stage, GATK-CombineGVCFs was utilized to combine the individual Genomic Variant Call Format (GVCF) files into a cohort GVCF. The calling of variants based on the cohort GVCF was accomplished using GATK GenotypeGVCFs. To ensure the inclusion of only high-confidence variants, GATK VariantFiltration was employed, applying rigorous filtering based on various quality metrics. BHK-derived and W8-derived viral variants were analyzed together against the NCBI SRA-generated reference. BHK-derived viral variants were analyzed against the W8-derived viral sequence consensus. Finally, the functional impact of the identified variants was elucidated using VEP from Ensembl to provide insights into the potential effects of variants on genes and proteins.

### 2.10. Data and Statistics

The minimum number of animals per group was determined based on pertinent literature for comparable studies in which the desired effect sizes for morbidity and mortality of infected and controls were shown to be statistically significant [5,7,8]. Daily samples collected from treatment and control groups were expected to be sufficient to observe and analyze differences in weights and viral titers in blood using paired *t*-test and survival curves using Mantel–Haenszel Hazard Ratios and Log-rank Mantel–Cox analyses. One-way analysis of variance (ANOVA) with Sidak’s multiple comparisons was used to compare Ct values and viral RNA concentrations in tissues. Statistical analyses were conducted using GraphPad Prism v.10.2 (GraphPad Software Inc., La Jolla, CA, USA).

## 3. Results

### 3.1. Clinical Disease

**Figure 1:**
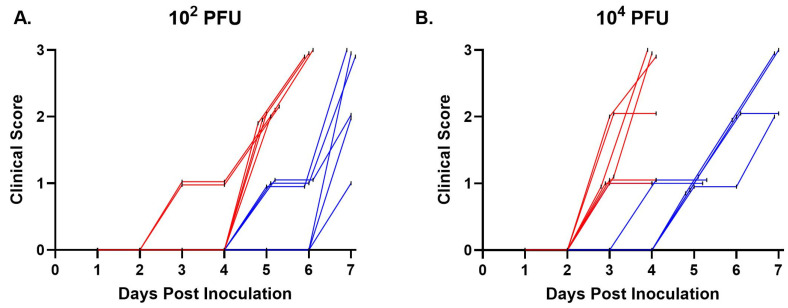
Clinical scores of IFNAR mice following inoculation with (**A**) 10^2^ PFU and (**B**) 10^4^ PFU BTV-17 derived from *Culicoides* W8 cells (red) or baby hamster kidney (BHK) cells (blue). Data points are XY staggered to show individual scores.

**Figure 2:**
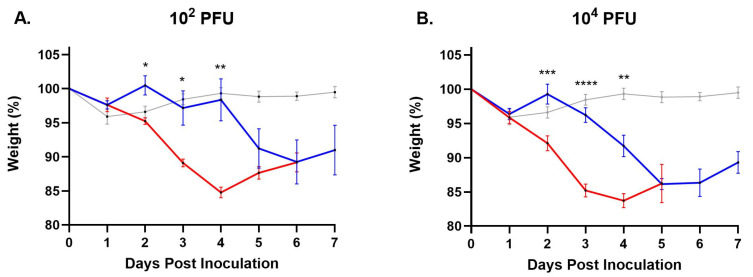
Mean body weights, as percent of starting weight, in IFNAR mice following inoculation with (**A**) 10^2^ PFU and (**B**) 10^4^ PFU BTV-17 derived from *Culicoides* W8 cells (red) or baby hamster kidney (BHK) cells (blue). Mean weights of mock-infected negative control mice are shown in gray. Error bars represent the standard error of the mean (SEM; n = 6). Paired *t*-test was used to determine statistical significance between W8 and BHK treatment groups as indicated (* *p* ≤ 0.05, ** *p* ≤ 0.01, *** *p* ≤ 0.005, **** *p* ≤ 0.001).

The overall disease parameters included daily weights, clinical disease onset and severity, viremia titers, tissue titers, and survival. Clinical signs were observed in all virus-inoculated IFNAR treatment groups. Following transdermal injection with the low dose (10^2^ PFU) virus inocula, clinical signs started two days earlier and peaked one day earlier in mice receiving W8-BTV-17 compared to BHK-BTV-17 (Figure 1A). In the high dose (10^4^ PFU) IFNAR groups, clinical signs started one day earlier and peaked three days earlier in W8-BTV-17 mice compared to BHK-BTV-17 mice (Figure 1B). Loss in body weight was significantly greater in mice inoculated with W8-BTV-17 compared to BHK-BTV-17 for both challenge doses on days 2, 3, and 4 (Figure 2), which corresponded to the onset and peak of clinical signs (Figure 1). No clinical signs were observed in the BTV-refractory C57BL/6J parental strain mice.

### 3.2. Survival 

**Figure 3:**
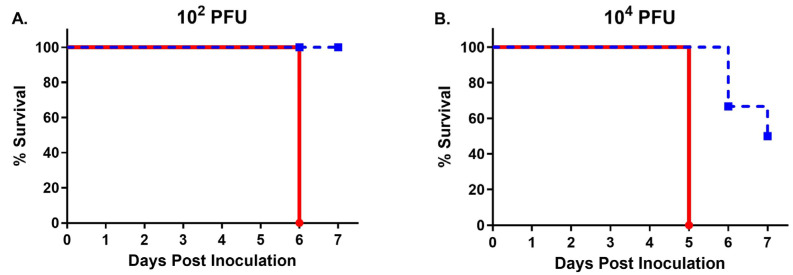
Survival curves of IFNAR mice following transdermal inoculation with BTV-17 derived from *Culicoides* W8 cells (red, solid) or baby hamster kidney (BHK) cells (blue, dashed). (**A**) Mice inoculated with the 10^2^ PFU low dose (Mantel–Haenszel Hazard Ratio = 41.03; Log-rank Mantel–Cox *p* = 0.0005). (**B**) Mice inoculated with the 10^4^ PFU high dose (Mantel–Haenszel Hazard Ratio = 39.12; Log-rank Mantel–Cox *p* = 0.0009).

Overall, W8-BTV-17 was significantly more lethal in IFNAR mice than BHK-BTV-17. The W8-BTV-17 low dose (10^2^ PFU) virus inoculum killed 100% of IFNAR mice by day 6 compared to 0% for BHK-BTV-17 by day 7 (Figure 3A), resulting in a 41 times higher rate of mortality (*p* = 0.0005). W8-BTV-17 at the high dose (10^4^ PFU) killed 100% of IFNAR mice by day 5 compared to 50% for BHK-BTV-17 by day 7 (Figure 3B), resulting in a 39 times higher rate of mortality (*p* = 0.0009). No mortality was seen in the BTV-refractory C57BL/6J parental strain mice.

### 3.3. Gross Pathology

**Figure 4:**
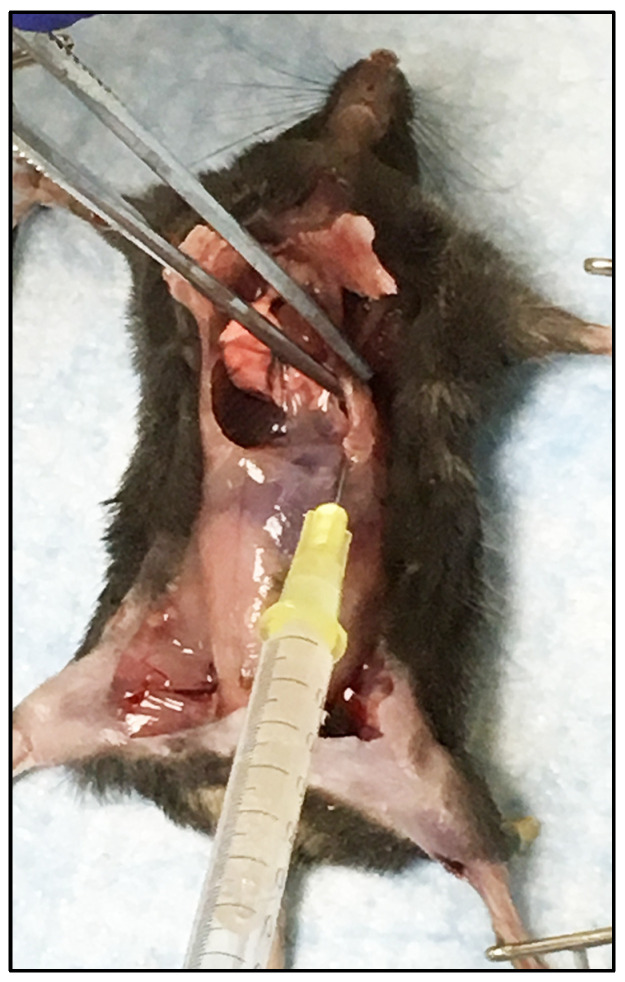
IFNAR mouse inoculated with 10^2^ PFU W8-BTV-17 euthanized 6 dpi due to a clinical score of 3. Severe edema (over 0.5 mL serosanguinous fluid as shown) in the thoracic cavity and wet abdominal serosal surfaces were observed in all IFNAR mice inoculated with both low (10^2^ PFU) and high (10^4^ PFU) dose W8-BTV-17 and in three inoculated with the high dose of BHK-BTV-17.

Necropsies were performed for tissue collection and gross pathology observations recorded for all IFNAR mice upon death or euthanasia (i.e., mice with clinical scores of 3 and all surviving mice at day 7). Gross pathology was consistent for all 12 W8-BTV-17 mice inoculated with the low dose (6 dpi) and the high dose (5 dpi), as well as the three high dose BHK-BTV-17 mice that were euthanized due to clinical scores or died on day 6 (n = 2) and 7 (n = 1). Inguinal and mesenteric lymph nodes were enlarged, and livers were slightly enlarged with variable sized white foci. Spleens were consistently enlarged and congested. Lungs were pale with petechial hemorrhaging on pleural surfaces and did not collapse after the thoracic cavity was opened. Severe edema was observed in the thoracic cavity with approximately 0.1 mL to 0.5 mL of serosanguinous fluid (Figure 4). Abdominal serosal surfaces were also very wet and edematous. The observed lesions suggest widespread endothelial damage and inflammation leading to tissue necrosis which is supported by the timing of clinical disease onset. Necropsies were performed on all BTV-refractory C57BL/6J parental strain mice with no pathology noted.

### 3.4. RNAemia

**Figure 5:**
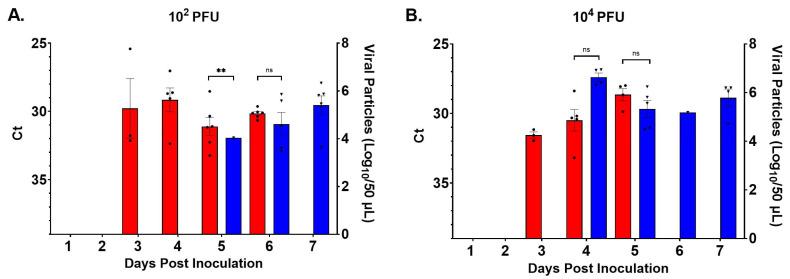
Mean virus titers in 50 µL daily blood samples as detected by RT-qPCR in IFNAR mice following challenge with (**A**) 10^2^ PFU or (**B**) 10^4^ PFU BTV-17 derived from either *Culicoides* W8 cells (red with dots) or baby hamster kidney (BHK) cells (blue with triangles). RT-qPCR cycle threshold (Ct; left Y-axis) and Log_10_ virus particle calculations (right Y-axis) based on RNA concentrations [39]. Error bars represent the standard error of the mean (SEM). Multiple paired *t*-test was used to determine statistical significance between W8 and BHK treatment groups as indicated (ns, not significant, ** *p* ≤ 0.01). Missing data for W8-BTV-17 inoculated mice was due to 100% mortality by day 6 (low-dose group) and by day 5 (high-dose group).

Mice infected with W8-BTV-17 at 10^2^ PFU had detectable RNAemia two days earlier than BHK-BTV-17-infected mice (Figure 5A). At the higher dose (10^4^ PFU), the onset of RNAemia in W8-BTV-17-infected mice was one day earlier than BHK-BTV-17-infected mice (Figure 5B). Due to the limited volume of daily blood collections, the entire 50 µL sample per mouse was used for RT-qPCR detection; the infectious virus was not titered by plaque assay. No viral RNA was detected in daily blood samples of the BTV-refractory C57BL/6J parental strain mice.

### 3.5. BTV-17 in Tissues

**Figure 6:**
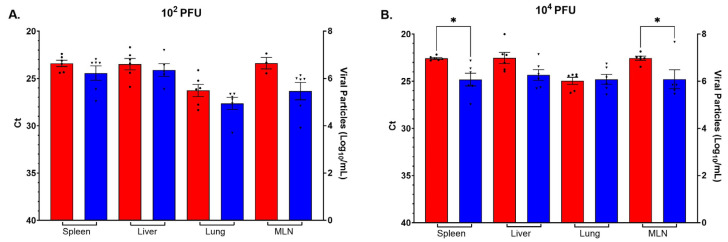
BTV-17 as detected by RT-qPCR in IFNAR mice necropsy tissue samples following challenge with (**A**) 10^2^ PFU or (**B**) 10^4^ PFU BTV-17 derived from either *Culicoides* W8 cells (red with dots) or baby hamster kidney (BHK) cells (blue with triangles). Mean RT-qPCR cycle threshold (Ct; left Y-axis) and Log_10_ virus particle calculations (right Y-axis) based on RNA concentrations [39] per mL of tissue homogenate. MLN; mesenteric lymph node. One way ANOVA with Sidak’s multiple comparisons was used to determine statistical significance as indicated (* *p* ≤ 0.05). Error bars represent the standard error of the mean (SEM, n = 6).

BTV-17 was detected by RT-qPCR in all tissue types taken at necropsy from at least three mice in each BTV-infected IFNAR group. The BTV RNA-positive tissues detected in all six mice in all groups were spleen, liver, lung, and mesenteric lymph node (Figure 6). No significant differences in viral RNA titers were seen between W8-BTV-17 and BHK-BTV-17 low-dose groups (Figure 6A). However, because all mice infected with W8-BTV-17 died or had to be euthanized due to a clinical score of 3 on day 6, RNA detected in these mice represents virus titers in tissues 1 day earlier than detected titers from BHK-BTV-17 mice, which were all euthanized at the end of the study (day 7). For the high-dose groups, significantly more viral RNA was detected in the spleen and mesenteric lymph node of mice infected with W8-BTV-17 compared to BHK-BTV-17 (Figure 6B). Because all mice infected with W8-BTV-17 died or had to be euthanized on day 5, titers represent viral RNA in tissues 1–2 days earlier than titers from tissues of BHK-BTV-17 mice, with two necropsied on day 6 and the remaining four on day 7. No viral RNA was detected in tissues of the BTV-refractory C57BL/6J parental strain mice.

**Figure 7:**
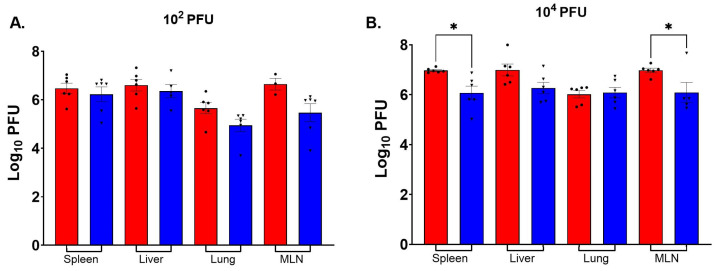
Infectious virus titers in tissues of IFNAR mice as detected by plaque assay following transdermal inoculation with (**A**) 10^2^ PFU and (**B**) 10^4^ PFU BTV-17 derived from either *Culicoides* W8 cells (red with dots) or baby hamster kidney (BHK) cells (blue with triangles). MLN; mesenteric lymph node. One-way ANOVA with Sidak’s multiple comparisons was used to determine statistical significance as indicated (* *p* ≤ 0.05).

Infectious BTV-17 was detected by plaque assay in all tissue types taken at necropsy from all BTV-infected IFNAR groups. The most predominant infectious virus-positive tissues were spleen, liver, lung, and mesenteric lymph node (Figure 6). No significant differences in virus titers were seen between W8-BTV-17 and BHK-BTV-17 10^2^ PFU low-dose groups (Figure 7A). However, as above, because all mice infected with W8-BTV-17 died or had to be euthanized on day 6, titers represent virus quantities in tissues 1 day earlier than titers in tissues of BHK-BTV-17 mice, which were all euthanized at the end of the study (day 7). For the 10^4^ PFU high-dose groups, significantly more virus was detected in the spleen and mesenteric lymph node of mice infected with W8-BTV-17 compared to BHK-BTV-17 (Figure 7B). This corresponds to the higher viral RNA quantities detected by RT-qPCR (Figure 6B). As above, because all mice infected with W8-BTV-17 died or had to be euthanized on day 5, the titers represent virus quantities in tissues 1–2 days earlier than titers from tissues of BHK-BTV-17 mice, with two necropsied on day 6 and the remaining four on day 7. No infectious virus was detected in tissues of the BTV-refractory C57BL/6J parental strain mice.

### 3.6. Sequence Analysis

Ensembl Variant Effect Predictor (VEP) analysis showed a total of 15 nucleotide substitutions common to both W8-derived BTV-17 and BHK-derived BTV-17 stocks originating from the 2007 MT/WY USA isolate [35] compared to the NCBI Sequence Read Archive reference strain (1988 CA USA) [41] (Table 3). Single nucleotide substitutions were found on all segments except segment 1 (VP1, RNA-dependent RNA polymerase) [42] and segment 4 (VP4, capping protein) [43]. All but two substitutions were found in gene coding sequences. No protein coding or secondary structure changes to proteins were predicted by VEP. For genome comparisons of the *Culicoides* W8-derived and BHK-derived viruses used to inoculate mice, only two nucleotide changes were found (Table 4). One in segment 2, which encodes the viral attachment protein VP2 [44], and one in segment 8 which encodes NS2, shown to play a role in virus replication and core assembly [45]. The substitution in segment 2 was a nonsynonymous transition at the stop codon of BHK-BTV-17 (TAG > CAG) resulting in a read-through until the second stop three codons later. The substitution in segment 8 was a synonymous transition in W8-BTV-17 (GLY > GLY) in the penultimate codon of the open reading frame. Neither substitution was predicted to change the proteins by VEP analysis. Therefore, the two nucleotide changes, which may have arisen from the different cell type environments, were not considered as contributing to the increased virulence of W8-BTV-17 seen in the IFNAR mice. 

## 4. Discussion

Experimental animal infections with BTV are critical to understanding pathogenesis, tissue tropism, transmissibility, and for the development of diagnostics, therapeutics, and vaccines. Natural, midge-transmitted BTV-17 outbreaks in U.S. sheep result in moderate to severe clinical disease and up to 35% mortality [35]. Yet, using high-titer BHK-derived viral inocula of this serotype and other established U.S. serotypes in experimental infections often results in only a mild, transient fever. The factors contributing to this vector-enhanced infection seen in midge-transmission remain unknown but likely relate to the route of viral delivery (intradermal), midge salivary proteins delivered with the virus [18,33], and the animal’s immune responses to the bite trauma and salivary proteins [34]. Although the goal of experimental animal studies is to mimic natural infections as closely as possible, conducting insect-transmission infection studies is logistically difficult and experimentally problematic. The addition of infected insects to a study design can quickly elevate biosafety and biosecurity risks and significantly complicate an experiment, whether in low- or high-containment facilities. Vector transmission studies are also experimentally problematic in terms of the infectious dose, due to the significant virus titer variability between individual insects and the inability to control the number of times an insect will probe before feeding to repletion, excreting saliva and virus each time. This results in highly variable infectious doses and an inability to obtain accurate biological replicates.

Because of these logistical and experimental complications, BTV transmission studies routinely utilize syringe-injection routes of inocula. Midge mouthparts typically penetrate the epidermis, entering the dermis of animal skin. Therefore, intradermal (ID) injection most closely mimics the site of insect-delivered virus during feeding and has been shown to result in early infection of gamma delta T-cells among other cells recruited to the bite site [34,46]. Here, the preferred method of virus delivery was ID; however, even with small-volume (5 uL) injections, it is extremely difficult to successfully achieve this in mice without some inocula going subcutaneous at some injection sites. Therefore, although ID was intended, transdermal is a more accurate description of mouse inoculations. In large animals, because transmission and subsequent infection are less efficient with injection than with natural vector-borne transmission, large volumes of high-titer inocula are typically injected, making unnatural routes of delivery (SC and IV) necessary, further compromising the goal of experimentally mimicking a “natural” infection.

Experimental inocula comprised of blood or washed blood cells from BTV-infected animals of the same or different species has been used successfully in animal infection studies [13,15,17,18,19]. However, obtaining properly processed, viremic blood from an infected animal can be logistically difficult and may contain additional contaminating pathogens. Additionally, BTV readily attaches to blood cells [47,48], which may compromise the attachment of virus to cells in the recipient. Therefore, high-titer cell culture-derived viral inocula are often used. BHK cells are highly susceptible to BTV and have been traditionally used to propagate high-titer stocks for animal infections [12,20,21,22,23,24,25,26]. Whether injecting blood or BHK-derived virus for these studies, the midge vector is removed completely from the inoculum production and the transmission/infection scenario.

We hypothesized that in addition to the physical trauma of the bite and the introduction of saliva, *Culicoides* proteins and the midge’s cellular environment in which the virus replicates before entering the mammalian host may also play a role in the efficiency of transmission and severity of infections observed in natural outbreaks. If so, a *Culicoides* cell line, derived from midge embryos and therefore containing cells from all midge tissues [36], could potentially be exploited to bring an in vitro vector-enhancement aspect to in vivo experimental animal infections. This would avoid the inherent logistical and experimental complications of live insect transmission and provide replicate animal challenge doses of midge-derived virus. 

Previous experimental studies with the highly virulent serotype BTV-8 [27,28,31], have used viral inocula derived from the original *Culicoides* cell line (KC) developed in 1989 [49]. However, severe clinical disease has been experimentally reproducible for this serotype, regardless of the virus source. Additionally, side-by-side comparisons of KC-derived virus with BHK-derived virus or viremic blood have not been reported to demonstrate whether the cell source made a difference in disease outcome for this virulent serotype in target animal species. In the BHK/W8 side-by-side comparison study described here, the only difference between the dose-equivalent treatments was the cell source of the virus. The results demonstrate an in vitro vector-enhancement of infection is possible in the BTV IFNAR mouse model by using midge cells to make the inoculum. IFNAR mice have been shown to have decreased IFN-α/β secretion, decreased IL-6 secretion, decreased TNF secretion and delayed NTK cellular functioning [7,50]. The typical onset of viremia and clinical signs within 48 h of inoculation strongly suggests a failure to elicit an effective innate immune response. Type 1 interferons activate many antiviral responses mediated through the Janus kinase/signal transducer and activator of transcription (JAK/STAT) pathways and are critical for the induction of adaptive immune responses. Ablated immune responses, early onset of clinical signs, and increased mortality have been observed in IFNAR mice after numerous RNA and DNA viral infections [51,52,53,54]. Here, BTV derived from *Culicoides* cells was significantly more virulent in IFNAR mice compared to the same stock virus grown in BHK cells. Mice inoculated with W8-BTV-17 had earlier onset of viremia, earlier onset and peak of clinical signs, and significantly higher mortality compared to mice inoculated with BHK-derived BTV-17 at the same titers. As no infectious virus or viral RNA was detectable in the blood or tissues of the C57BL/6J mice, the enhanced infection of the W8-BTV-17 was not significant enough to overcome the refractory nature of this parental strain. Therefore, IFNAR mice remain the only BTV-susceptible adult murine model system, regardless of inoculum source [7,50].

Comparative analyses of the consensus genome sequences obtained from the W8-BTV-17 and BHK-BTV-17 showed no predicted changes to viral proteins. Therefore, genome mutations were not considered as contributing to the increased virulence seen in IFNAR mice. Nonetheless, selective pressures on virus replication in the two environments would be different, as would glycosylation of the outer capsid protein VP5, which is important for cell penetration [55,56]. In vitro BTV infections of midge cells result in a chronic, persistent, non-lytic infection, whereas BTV infection of mammalian cells (BHK, bovine, ovine) results in an acute, lytic infection. Higher genome-wide genetic diversity has been observed in chronic versus acute infections with other viruses [57,58,59]. The degree of genetic diversity in BTV populations arising from *Culicoides* cells compared to mammalian cells has been proposed as a possible source of virulence [60,61]. Yet, similar variant diversity has been reported for some genes when BTV is cultured solely in *Culicoides* or bovine cells or when alternating host–cell types [41,62]. A preliminary analysis of variants using the sequence data obtained in this study showed a trend toward increased minority variants and therefore greater genetic diversity in viral populations of the *Culicoides*-derived BTV compared to the BHK-derived BTV, but deep sequencing efforts would be needed to further elucidate increased diversity in *Culicoides*-derived BTV in relation to virulence. 

Cleared, freeze-thawed, infected cell lysates are routinely used to make BTV inocula for experimental animal infections. In this study, viral stocks for mice inoculations were purposely made as viral lysates, not purified virus, to be consistent with how BHK-BTV inocula are typically produced. BHK cellular proteins in these traditional inocula have been shown to cause hypersensitivity in some vaccine trials [63,64,65]. The BHK and W8 viral lysate stocks were produced identically to ensure any changes in virulence would be attributed to both the virus and any cellular proteins typically found in a lysate infection inocula. It is possible that midge cell proteins in the cleared lysate of W8-BTV-17 could specifically inhibit or delay mammalian adaptive immune responses [66], resulting in a faster infection (earlier onset of viremia and clinical disease) and/or enhance immunopathology (increased mortality). Although the full genome reference sequence of adult female *Culicoides sonorensis* is available [67], annotation is ongoing, and other than saliva [33], proteomes are not yet available to further elucidate this possibility. 

For this and many BTV serotypes, high-titer BHK-derived viral inocula, in large dose volumes requiring unnatural routes of delivery are used to obtain clinically infected animals representative of those seen in natural midge-borne infections. Overall, our results show that using a *Culicoides* W8 cell lysate inoculum in the BTV IFNAR mouse infection model adds a midge vector component to the experimental scenario and enhances the virulence compared to BHK cell lysate inocula. This may represent an in vitro vector-enhancement methodology for laboratory and target-animal infection studies with this and other BTV serotypes while avoiding the logistically and experimentally problematic complexity of working with live midges.

## Figures and Tables

**Table 1:** BTV-17 inoculations of C57BL/6J and IFNAR mice.

Treatment Groups	Inocula	N
Mock		
C57BL/6J	Cleared BHK cell lysate	3
IFNAR	Cleared BHK cell lysate	3
C57BL/6J	Cleared W8 cell lysate	3
IFNAR	Cleared W8 cell lysate	3
BHK-BTV-17 Low Dose		
C57BL/6J	10^2^ PFU BHK-BTV-17	6
IFNAR	10^2^ PFU BHK-BTV-17	6
W8-BTV-17 Low Dose		
C57BL/6J	10^2^ PFU W8-BTV-17	6
IFNAR	10^2^ PFU W8-BTV-17	6
BHK-BTV-17 High Dose		
C57BL/6J	10^4^ PFU BHK-BTV-17	6
IFNAR	10^4^ PFU BHK-BTV-17	6
W8-BTV-17 High Dose		
C57BL/6J	10^4^ PFU W8-BTV-17	6
IFNAR	10^4^ PFU W8-BTV-17	6

**Table 2:** Scoring and characterization of clinical bluetongue disease in IFNAR mice.

Score	Clinical Signs	Characterization
0	None	Bright, alert, responsive
1	Mild	Ruffled fur, mild nasal exudate, ocular discharge
2	Moderate	Mild plus: lethargy, hunched posture, moderate nasal exudate, moderate ocular discharge, labored respiration
3	Severe	Moderate plus neurological signs: paralysis, tremors, ataxia, rigidity, coma

**Table 3:** Variant effect predictions of W8-BTV-17 and BHK-BTV-17 compared to NCBI reference genome. Both virus stocks were compared to BTV-17 USA 1988/CA reference genome from the NCBI Sequence Read Archive. Allele change identical in both W8 and BHK stocks.

Accession	Segment	Gene	Location	Coding Region	Reference NT	Allele
MT952972.1	2	VP2	2291 2892	20-2887	A T	G C
MT952973.1	3	VP3	662 932 1221 1716	18-2723	C A T A	T G C G
MT952975.1	5	NS1	377 708	23-1681	A T	G G
MT952976.1	6	VP5	1443 1624	30-1610	G A	A G
MT952977.1	7	VP2	708	18-1067	C	T
MT952978.1	8	NS2	591	20-1084	A	G
MT952979.1	9	VP6	30 56	16-1005	G A	A T
MT952980.1	10	NS3	360	19-708	A	G

**Table 4:** Variant effect predictions comparing genomes of W8-BTV-17 and BHK-BTV-17 mouse inocula.

Accession	Segment	Gene	Location	Coding Region	Reference NT	W8 NT	BHK NT
MT952972.1	2	VP2	2885	20-2887	T	T	C
MT952978.1	8	NS2	1078	20-1084	T	C	T

## Data Availability

The data presented in this study will be made available on Ag Data Commons and are available by request from the authors.

## Supplementary Materials

The following supporting information can be downloaded at https://www.mdpi.com/article/10.3390/v16091474/s1, Table S1: BTV-17 reference genome.
