# Supplementary material for: Increased Virulence of Culicoides Midge Cell-Derived Bluetongue Virus in IFNAR Mice
Source: Viruses. 2024 Sep 17;16(9):1474. doi: 10.3390/v16091474 (PMC11437402; doi:10.3390/v16091474)
Supplement: Supplementary file 1 [file viruses-16-01474-s001.zip › viruses-3187041-supplementary.pdf]

Table S1. BTV-17 reference genome. GenBank gene accessions used to create the BTV-17 reference genome for comparisons to BHK and W8 viral stocks. Illumina-derived sequences were concatenated into a single file and GFF calculated from combined sequences. Bowtie v.2 (John Hopkins University) was used for all original assemblies.

Accession	Virus	Segment	Source Link	Definition
MT952971.1	BTV-17	1	www.ncbi.nlm.nih.gov/nuccore/MT952971.1	Bluetongue virus isolate BTV-17 USA1988/CA segment 1 complete sequence
MT952972.1	BTV-17	2	www.ncbi.nlm.nih.gov/nuccore/MT952972.1	Bluetongue virus isolate BTV-17 USA1988/CA segment 2 complete sequence
MT952973.1	BTV-17	3	www.ncbi.nlm.nih.gov/nuccore/MT952973.1	Bluetongue virus isolate BTV-17 USA1988/CA segment 3 complete sequence
MT952974.1	BTV-17	4	www.ncbi.nlm.nih.gov/nuccore/MT952974.1	Bluetongue virus isolate BTV-17 USA1988/CA segment 4 complete sequence
MT952975.1	BTV-17	5	www.ncbi.nlm.nih.gov/nuccore/MT952975.1	Bluetongue virus isolate BTV-17 USA1988/CA segment 5 complete sequence
MT952976.1	BTV-17	6	www.ncbi.nlm.nih.gov/nuccore/MT952976.1	Bluetongue virus isolate BTV-17 USA1988/CA segment 6 complete sequence
MT952977.1	BTV-17	7	www.ncbi.nlm.nih.gov/nuccore/MT952977.1	Bluetongue virus isolate BTV-17 USA1988/CA segment 7 complete sequence
MT952978.1	BTV-17	8	www.ncbi.nlm.nih.gov/nuccore/MT952978.1	Bluetongue virus isolate BTV-17 USA1988/CA segment 8 complete sequence
MT952979.1	BTV-17	9	www.ncbi.nlm.nih.gov/nuccore/MT952979.1	Bluetongue virus isolate BTV-17 USA1988/CA segment 9 complete sequence
MT952980.1	BTV-17	10	www.ncbi.nlm.nih.gov/nuccore/MT952980.1	Bluetongue virus isolate BTV-17 USA1988/CA segment 10 complete sequence
